# Human-centered design for global health equity

**DOI:** 10.1080/02681102.2019.1667289

**Published:** 2019-09-29

**Authors:** Isaac Holeman, Dianna Kane

**Affiliations:** a Department of Global Health, The University of Washington, Seattle, WA, USA; b Medic Mobile, Seattle, WA, USA

**Keywords:** Digital health, global health equity, human-centered design, ICT4D, participatory design, user-centered design, mHealth, co-design, design thinking, eHealth, implementation research

## Abstract

As digital technologies play a growing role in healthcare, human-centered design is gaining traction in global health. Amid concern that this trend offers little more than buzzwords, our paper clarifies how human-centered design matters for global health equity. First, we contextualize how the design discipline differs from conventional approaches to research and innovation in global health, by emphasizing craft skills and iterative methods that reframe the relationship between design and implementation. Second, while there is no definitive agreement about what the ‘human’ part means, it often implies stakeholder participation, augmenting human skills, and attention to human values. Finally, we consider the practical relevance of human-centered design by reflecting on our experiences accompanying health workers through over seventy digital health initiatives. In light of this material, we describe human-centered design as a flexible yet disciplined approach to innovation that prioritizes people's needs and concrete experiences in the design of complex systems.

## 1. Introduction

In 2011 the World Health Organization proclaimed that mobile technologies have the ‘potential to transform the face of health service delivery across the globe’ (Kay, Santos, & Takane, 2011, p. 1). More than 96% of the world's population now lives within reach of a mobile phone signal (Sanou, 2015) and studies have demonstrated that this infrastructure can be used to measurably improve health outcomes (Lester et al., 2010) and to strengthen the health workforce (Zurovac et al., 2011). Labrique, Vasudevan, Kochi, Fabricant, and Mehl (2013) make a compelling case for viewing mobile and web technologies as health systems strengthening tools, enabling new ways of coordinating, decentralizing and expanding the quality and equity of care.

Nonetheless, numerous researchers have registered concern with the poor scalability or reproducibility of successes, using the term ‘pilotitis’ to suggest that this plagues digital health efforts in lower-income settings (Shuchman, 2014; Tomlinson, Rotheram-Borus, Swartz, & Tsai, 2013; Waugaman, 2016). Too many mHealth projects falter due to simplistic assumptions about end user preferences and activities, or because large-scale implementations are far more complex than small trials. Digital technologies evolve even as they are implemented, as does the process by which they are delivered. This is not only because technologies advance rapidly, but also because stakeholders often reasonably demand changes in order to integrate multiple health programs or to accommodate local infrastructure and health worker routines. Such complexity and rapid change often plays out amid the conflicting priorities of myriad governmental and non-governmental decision-makers, resulting in the sort of messy challenges that design theorists call *wicked problems* (Buchanan, 1992; Rittel & Webber, 1973). These complexities are not unique to technology innovation; many implementers of global health programs are familiar with such challenges because they surface often in efforts to strengthen health systems in hard-to-reach communities. As recent calls for global health implementation research attest (Kim, Farmer, & Porter, 2013; Kruk et al., 2016), delivering equitable healthcare that actually reaches poor people is a wicked problem of great human consequence.

In 2008 we and our colleagues established a novel intervention for coordinating community health workers (CHWs) through conversational text messaging (Holeman et al., 2014; Mahmud, Rodriguez, & Nesbit, 2010). This effort gave rise to an open source project called the Community Health Toolkit, and to the non-profit organization Medic Mobile, with which we are both affiliated. Despite our initial successes in small pilots, efforts to scale-up our work in partnership with a wider array of health system stakeholders were beset by numerous implementation difficulties, many of which could only be meaningfully addressed by redesigning our interventions. Through these experiences, we began to see pilotitis in digital health as a symptom of the broader implementation complexities that make global health equity a wicked problem. We realized that it is often impractical or even amateurish to replicate evidence-based technological interventions in an inflexible ‘cookie-cutter’ manner, let alone to trust that outcomes would be similar to those observed in prior trials. This led us to cultivate a more flexible, contextually-driven process for designing complex systems. By 2010 human-centered design had become central to Medic Mobile's approach to innovation for global health.

For the first few years of Medic Mobile's work, human-centered design was a largely unfamiliar term in the global health community; justifying resources and dedicating ample time for it was usually difficult. However, growing attention to implementation challenges and the rise of digital technologies in care delivery have fostered interest in what constitutes rigorous design practice. Part of the appeal of focusing on the design process is that it recognizes the potential of tech-enabled innovation, without attributing impacts narrowly to particular technologies or technical fixes (Holeman & Barrett, 2017). Since 2012 dozens of authoritative global health institutions including USAID, The Bill and Melinda Gates Foundation, and several United Nations agencies have elevated this conversation by endorsing nine design principles for digital development (Waugaman, 2016). How-to guides on human-centered design for global health and social innovation have proliferated (Kimbell, 2014),^1^ as have news articles and white papers documenting the design experiences of healthcare organizations (Artefact Group, 2014; USAID, 2016; Veterans Affairs, 2014).

We see this as a valuable development, yet there is concern in some circles that human-centered design has become a sort of craze, and may be just another feel-good, global health and development buzzword (Cheney, 2016; Guardian, 2015; Lee, 2015a; Lee, 2015b). Popular guides and case studies typically cite few if any resources for further reading in the rigorous academic literature on design. As this paper will show, many now use the term without reflecting critically on its implications for widely accepted models of medical evidence and practice.

In light of this trend, the primary aim of our paper is to clarify how human-centered design may be of value to the scientific and practical agenda of global health equity. This is not a purely academic exercise, but part of an ongoing action research effort focused on supporting a growing community of designers, developers, and implementers of the Community Health Toolkit. Taking inspiration from DHIS2, OpenMRS, and other communities that support open source health information systems as global public goods, we are acutely aware that local teams typically depend on cooperation with related efforts in other settings. The promise of such a *network of action,* to use Braa, Monteiro, and Sahay’s (2004) term, is that global scaling enables cross-pollination of design and implementation practices, which can improve the sustainability of tech-enabled health systems strengthening efforts. With the needs of this growing community in mind, our paper begins with a summary of our research context and methods (section 2), and then draws on a combination of literature review and reflection on action research efforts to develop three contributions.

First, in section 3 we contextualize how the design discipline differs from more common approaches to research and innovation in global health. We emphasize craft skills that foster hands-on engagement, as well as iterative methods that reframe the relationship between design and implementation. Examining these themes is an important opportunity to make this work accessible to practitioners and to scientists who are new to design research, given that contemporary scholarship on human-centered design often takes these central features of design practice for granted.

Second, in section 4 we examine how design researchers distinguish ‘human-centered’ approaches from other kinds of design practice. We consider related yet distinct terms such as user-centered design and design thinking, and emphasize the diversity of perspectives within this multidisciplinary field. While there is no definitive agreement about what the ‘human’ part means, among design researchers it often implies stakeholder participation, a commitment to supporting or augmenting people's skills, and attending to human values in broader social and organizational context. These priorities make human-centered design distinctive, and they also make it highly relevant to health equity.

Finally, in section 5 we consider the practical relevance of human-centered design by reflecting on our own experiences with over seventy digital health initiatives over the last ten years. This section describes some challenges and outlines our view of human-centered design as a practical way of accompanying health workers and communities in their struggles for health equity. In this view human-centered design is not limited to building technologies or solving purely technical problems, so much as it is a way of making sense of the complex challenge of health systems strengthening in a digital age.

## 2. Research methods

The first part of this section introduces the concept of health equity and why it merits our attention. Next, we explain how we identified and analyzed the literature we summarize in sections 3 and 4. Finally, we offer some historical context on design at Medic Mobile, and present our process and reasons for undertaking the action research that informs section 5.

### 2.1. Research context: why study global health equity and human-centered design?

Health equity has always been central to Medic Mobile's work and to the Community Health Toolkit's design agenda. To understand why health equity matters for design and for global development, it is important to recognize that around the world there is a clear gradient: the higher a person's social status and economic means, the lower their mortality rate. As recently as 2004 there was a remarkable 48 year gap in life expectancy between Japan and Sierra Leone (WHO, 2004). Within the United States and Australia, there are life expectancy gaps of twenty years between the wealthiest and most marginalized groups (Marmot, 2005). This gradient is driven not only by acute material deprivation, but also by access to care. For example in Nigeria only 10% of the poorest and almost 80% of the wealthy have access to healthcare (WHO, 2015). Social conditions that increase the risk of non-communicable afflictions such as coronary heart disease play a role, through limited access to healthy food or decent housing, through unhealthy behaviors and unsafe workplaces, and through the effects of impossibly stressful lives (Marmot, 2004). Increased exposure to (or limited protection from) violence, humanitarian crises, and human rights abuses also figures into this trend (Farmer, 2004).

Such staggering differences in life expectancy and burden of disease are by no means inevitable. As Whitehead's (1991) classic definition puts it, ‘the term inequity has a moral and ethical dimension. It refers to differences which are unnecessary and avoidable but, in addition, are also considered unfair and unjust.’ The World Health Organization's commission on the social determinants of health makes a similar point, ‘if systematic differences in health for different groups of people are avoidable by reasonable action, their existence is, quite simply, unfair. We call this imbalance health inequity’ (Marmot, Friel, Bell, Houweling, & Taylor, 2008, p. 1661). That commission also emphasized that inequities in health matter for social and human development at large:

There is no difficulty in convincing medical and health personnel that health is important – that is what we do. It is more challenging, but necessary, to convince policy makers and others that the health of the population is important precisely because it is a measure of whether, in the end, a population is benefiting as a result of a set of social arrangements. (Marmot, 2005, p. 1103)

Thus health equity matters in its own right, and it also is a widely-recognized indicator of whether societies are meeting basic human needs (Sen, 1995). A growing number of ICT for development researchers are attending to health equity in their design and research efforts (Qureshi, 2016). To meaningfully influence practice, these studies will need to address the complex implementation challenges that lead so many promising digital health pilots to falter before replication or scale-up. Human-centered design is an approach to dealing with such challenges practically and with care; this is why human-centered design for global health equity merits our attention.

### 2.2. Locating and reviewing the academic design literature

In recent years the popular literature on design thinking and human-centered design has sparked growing interest in fields that had little prior engagement with this tradition of design research and practice, including public health and medicine, management, public policy, and development studies. Books such as Change by Design (Brown, 2009) and The Design of Business (Martin, 2009) and articles such as Design Thinking for Social Innovation (Brown & Wyatt, 2010) have communicated design issues to a wider public, though it is generally understood among design researchers that such popular writing should not be mistaken for the rigorous primary literature (Bannon & Ehn, 2013; Bjögvinsson, Ehn, & Hillgren, 2012; Kimbell, 2011; Tonkinwise, 2016).

Our initial attempt to undertake a systematic review of this literature proved unenlightening, in part because the relevant research is spread across several disciplines that historically have not used design-related terms in a coherent way. Some context on the research communities that our analysis draws from is important for understanding why we eventually embraced a less structured review of related work. A great deal of design research documents the design of particular artifacts, while some concerns the nature of design activities. Many associate the classic view of designing as a complex human activity with seminal works for example by Rittel and Webber (1973), Schön (1983), Krippendorff (1989), Buchanan (1992), Cross (2001), and Nelson and Stolterman (2003). This literature is well represented in journals such as Design Issues and Design Studies, the proceedings of the Design Research Society conferences, and the MIT Press series of books on design theory/design thinking. A complementary body of work is less associated with the design profession, but treats design as an important topic within other disciplines such as anthropology (Suchman, 2011) and science and technology studies (Latour, 2008).

The terms human-centered design, human-centered computing, and human-centered systems first came into widespread use in the last three decades,^2^ initially among computing and information systems researchers (Bannon, 2011; Kling & Star, 1998). Academic engineering conferences such as Computer–Human Interaction and Computer Supported Cooperative Work are important venues for such research. The psychological experiments that inform user-centered design and the related idea of user-friendliness (Norman, 2013) and the more recent but growing body of work on value-sensitive design (Friedman, 1996) are well represented in these venues. Design research including the participatory design and sociotechnical systems design traditions has been published at these engineering conferences, in information systems journals (Porra & Hirschheim, 2007; Sein, Henfridsson, Purao, Rossi, & Lindgren, 2011) and at dedicated outlets such as the bi-annual Participatory Design conference and the journal CoDesign. Since 2011 the Design for Health journal and associated conferences have produced a growing body of salient work. Of particular relevance for global health is the field of Information and Communication Technology for Development (ICT4D). According to relevant literature reviews (Dell & Kumar, 2016; Ho, Smyth, Kam, & Dearden, 2009; Walsham, 2017) this work is well represented in journals such as Information Technology for Development, publications from the ICTD and COMPASS conferences, and the larger engineering conferences mentioned above.

In medicine and public health it is commonplace to ignore this broader design literature, most of which is not indexed in PubMed, or to primarily reference popular design books and toolkits in lieu of academic design research.^3^ While references to such work are found more often in specialist journals such as the Journal of Medical Internet Research and Journal of the Medical Informatics Association, there is nonetheless a clear trend. The academic design literature is so vast, heterogeneous, or jumbled that when health researchers (not to mention practitioners) seek an accessible introduction, they are often forced to rely on popular guides that make little effort to cite sources or observe basic standards or scholarly rigor. This situation highlights the need for a broadly accessible literature review, while also raising methodological challenges.

To examine the relevance of human-centered design for global health equity, we needed to explore the transfer of ideas and practices across these research communities. We needed to be able to draw connections across conceptually related works even when they appeared superficially different (e.g. using different terms or taking place in different empirical contexts), while also being able to differentiate fundamentally distinct projects even when they used overlapping terms (e.g. the wide range of practices that now invoke the term ‘human-centered’). The fact that ‘design’ is a ubiquitous term in the English language did not make our search easier. Restricting our search to uses of the term ‘human-centered design’ would have excluded the foundational design research that preceded use of the term ‘human-centered’ and eventually came to shape it (e.g. sociotechnical systems research). The common strategy of limiting a systematic search to a specific database (e.g. PubMed) or a recognized set of leading journals in a single field would have been obviously problematic given this paper's concern with where human-centered design comes from and why it has garnered attention in the global health community. While systematic review methods (Liberati et al., 2009) are the norm in medicine and public health, all of these challenges led us to a conclusion that has been recognized elsewhere (Bates & Glennerster, 2017) – such reproducible methods break down and are less useful for reviewing highly ambiguous topics.

For these reasons, we chose to write a selective review of this literature in essay format, as is common across the social sciences including in ICT4D research (e.g. Walsham, 2017). This means our review process is not reproducible and our inclusion of some papers and not others inevitably involved a degree of subjective judgment. Bearing these limits in mind, we employed four strategies to develop a thorough picture of this literature: (1) reading available literature reviews (e.g. Bazzano, Martin, Hicks, Faughnan, & Murphy, 2017; Kling & Star, 1998) and essays on how the design discipline has evolved in recent decades (Bannon, 2011; Bannon & Ehn, 2013); (2) snowball sampling by examining references lists, and recent citations of highly cited ‘classic’ works; (3) sharing drafts with colleagues for friendly review (this paper was read by more than twenty researchers and dozens of students); and (4) relying on the process of peer-review to establish a baseline of thoroughness and rigor. Our highly iterative process for synthesizing this literature involved drawing selectively from publications across each of the research communities outlined above, writing initial drafts, seeking feedback, undertaking further reading, and re-writing. We would emphasize that feedback from colleagues and our editors and anonymous reviewers proved enormously helpful in identifying gaps in our analysis. For the sake of brevity and readability, this paper does not reference every single article and book that we read throughout the five-year period during which we wrote more than three dozen versions of this manuscript. Rather than attempting to comprehensively reference the most recent publications in every sub-field and recently developing stream of design research, we have emphasized highly cited, classic works that represent important developments in design scholarship. Our aim is not to be exhaustive, but to orient the curious researcher to pursue further exploration on their own.

### 2.3. Action research and human-centered design at Medic Mobile

In a further attempt to humanize the analysis and help readers relate conceptual design issues to concrete global health challenges, we also offer reflections on example projects and ongoing challenges drawn from our experiences practicing design at Medic Mobile. Formed in 2010 as a non-profit organization, Medic Mobile builds open source technology to improve healthcare in hard-to-reach communities. While recognizing the growth of mobile networks as an important opportunity, we were first and foremost inspired by community-based health workers who stand for the health of their neighbors, often with remarkable effort, humility, and courage. The people we met in Malawi, Kenya, Nepal and a growing number of countries generously shared their stories and gave us our first design principles: start with people not with tech, design for the familiar, address practical challenges from day one, and make room for big ideas that may take years of iteration to mature.

For this paper, we adopted action research methods to accommodate the long term, global, and community-driven (rather than researcher-driven) nature of this effort. Since 2008, we have participated in more than 70 digital health initiatives, and the Community Health Toolkit we designed has grown to support health workers that cover roughly 12 million people (see Table 1 for timeline of key implementation events). During this period, our command of human-centered design concepts and methods has advanced considerably through sustained reading, reflection, and practice. Research has played a fundamental role; Medic Mobile staff and our partners have produced more than thirty peer-reviewed publications. Study methods have ranged from ethnography (Holeman & Barrett, 2017) to randomized controlled trials (Whidden et al., 2018), and design research methods including research through design (Zimmerman, Forlizzi, & Evenson, 2007) and action design research (Sein et al., 2011). While each of these studies answered questions about specific design projects, over time we came to see that this collection of articles did not amount to an accessible, substantive reflection on our design practice as a whole – a gap we hoped to address through this paper.

**Table 1.** Timeline of key implementation events.

2008	First pilot of interactive text messaging for care coordination among community health workers established in Malawi. Made use of an open source tool called FrontlineSMS.
2009	Replication projects started elsewhere in Malawi and in Uganda, spreading through word of mouth, blogging, and volunteer effort.
2010	Medic Mobile incorporated as a non-profit, started design team, and implementation expanded to ten countries. First research paper on results of initial pilot was published.
2011	Work with 25 partners covered 1 million people. Medic Mobile began developing its new open source software framework for community health.
2012	Medic Mobile opened offices in Nairobi and San Francisco, established a research team, and began first randomized controlled trials. Programs covered 2 million people.
2013	A regional office opened in Kathmandu to serve projects across south Asia. After equipping 1500 additional health workers, programs covered 3.5 million people.
2014	Software expanded from messaging and task management into decision support, health records, and analytics. Skoll award for social entrepreneurship funded major growth.
2015	Medic Mobile played a role in Nepal earthquake response, and began implementing new Android app. Staff grew to 55 people and implementation footprint expanded by 50%.
2016	Expanded to 6800 new users, launched apps for nurses and supervisors. Transitioned from case-based to longitudinal records with better support for people-centered, integrated care.
2017	Deepened partnerships with governments in Kenya and Nepal. Launched Standard package to boost accessibility, and established staff presence in Kampala, Dakar.
2018	Medic Mobile and partners expand support for their open source community by launching new Community Health Toolkit (CHT) resources at communityhealthtoolkit.org.
2019	Software supports over 1 million home visits per month in health systems that cover 12 million people. About 100 Medic Mobile staff work in Nairobi, Kathmandu, Kampala, Dakar, San Francisco, Seattle, 30% work remotely. New initiatives focus on training designers and developers within governments and other organizations implementing the CHT.

The example of DHIS2 development by the HISP network (Braa et al., 2004) demonstrates that action research is amenable not only to short-term studies with clear phases, but also to long term, global open source communities characterized by ‘multiplicity and simultaneity of ongoing processes which take on different forms at various stages, and there is rarely ever a clear start or end’. Like Braa et al. (2004), we and our co-workers are continuously engaged in various design and implementation activities and we found that it would be impossible to adopt a singular and well-defined process of analysis or give quantitative details about the number of interviews conducted in the course of the last decade. To write this paper, we (one embedded researcher and one design leader) began by assembling over eighty documents including articles, internal reports, and conference presentations that described specific design projects or the design process overall at Medic Mobile. We drew on our long term practical experience with this initiative to synthesize these documents and begin writing. We iterated on initial drafts in light of conceptual themes that emerged in the literature review and by drawing extensively on feedback from our community of practice. Every major draft of the paper was circulated among Medic Mobile's designers, our research partners, and students at the University of Cambridge, the University of Edinburgh, and the University of Washington.

The findings of this process are presented in section 5, which aims to demonstrate the practical relevance of the design literature that we review in the first part of the paper. An important limitation of action research is that findings may lack the objectivity of a more reproducible analytical process in which researchers maintain greater distance from the institution being studied. Nevertheless, we hope that these transparent reflections will offer our readers greater perspective on our standpoint and how it shaped our review of the literature. More concretely, by integrating literature review and reflections on practical relevance, we hope that these findings will prove helpful to a growing community of designers and implementers of open source software for global health.

## 3. How does design differ from and complement other approaches in global health?

For the global health practitioner, it would be myopic to dwell on differences among different types of design practice, if we did not recognize that there exists a wider gulf between design generally and more prevalent approaches in global health. According to the Design for Health initiative, ‘design is a craft and a discipline that applies a specific mindset and skillset to a creative problem solving process’.^4^ The design mindset or attitude (Amatullo, 2015; Boland, 2004; Michlewski, 2016) often contrasts with how outsiders perceive the role for design, as this Steve Jobs quote suggests:

People think it's this veneer – that the designers are handed this box and told, 'Make it look good!' That's not what we think design is. It's not just what it looks like and feels like. Design is how it works.^5^

Design education also emphasizes apprenticeship in craft skills, such as the ability to sketch out or model an image of a work product to aid the process of actually building it, and deep intuitive familiarity with particular materials such as cloth, clay, paint, or steel. The workaday life of designers can vary greatly, given the diverse materials and circumstances of their work. Nonetheless, by observing the complex and often idiosyncratic way that designers think and work, researchers have derived an understanding of common patterns in design practice. Since at least the 1960s, researchers have used the term *design thinking* in reference to these theories of how accomplished designers think and work (Archer, 1965). The expression's popularity has grown over the decades with publications such as the book *Design Thinking* (1987) by Peter Rowe, dean of Harvard's graduate school of design.

**Figure 1.**
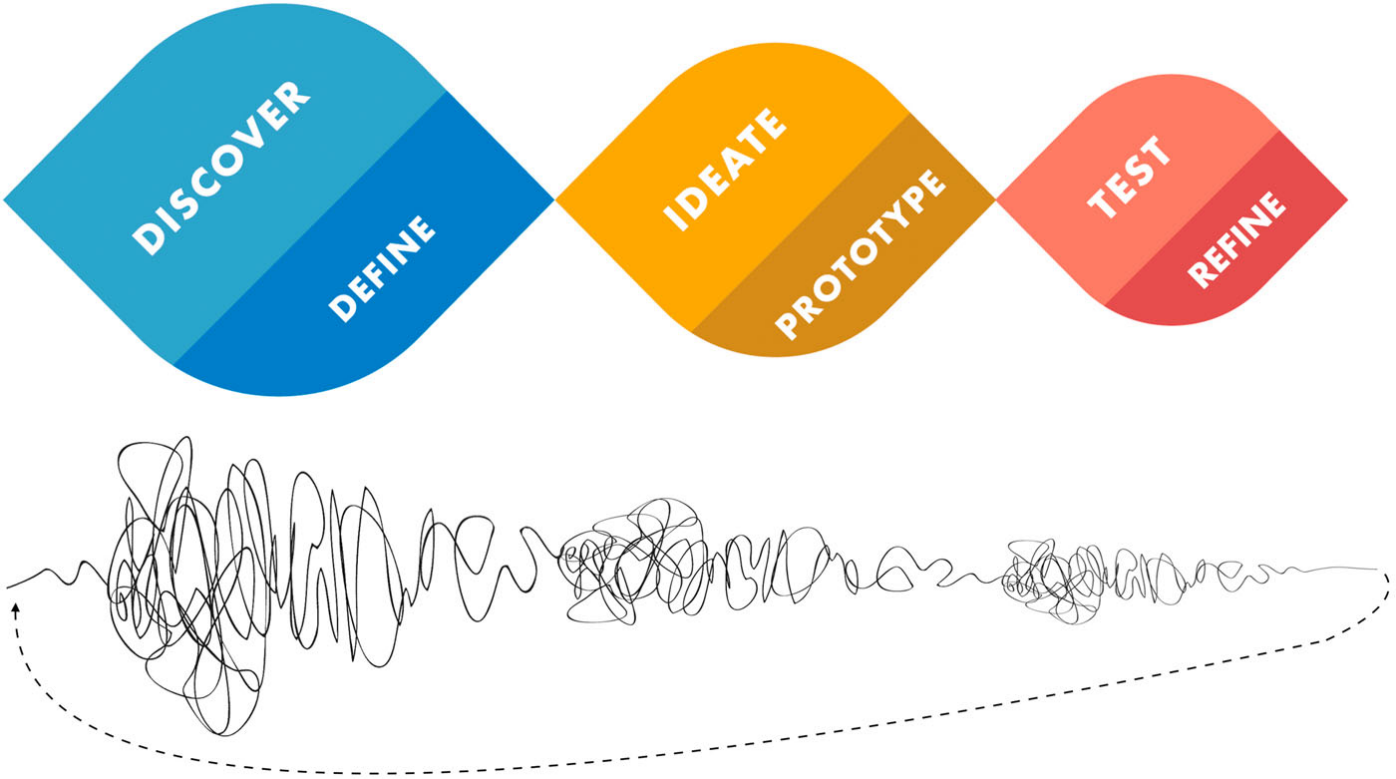
Two visuals of the design process.

Drawing on these insights, a number of more recent ‘how-to’ guides offer resources for beginners, introducing a design process that is broadly aligned with the visuals in Figure 1. Proponents of these practical resources hold that understanding how designers think and work is of value to people who are not professional designers, including organizations pursuing innovation and societies looking for creative approaches to solving intractable social problems. Some design professionals and researchers have critiqued the popular and commercial rhetoric for overpromising, or for offering only simplistic ‘design-lite’ perspectives on how expert designers actually work (Kimbell, 2011). We nonetheless see the growing appreciation of practical design guides as a promising development in global health, a field whose researchers and practitioners often find design language and practices startling, and may perceive them as disruptive or lacking in rigor if they are not explained well. With this audience in mind, the following outline is emphatically not intended as a strict conceptual definition of design thinking (in the design theory sense).^6^ Neither are these lessons intended as a cheap substitute for the kind of extended education and apprenticeship that design professionals undertake. Rather, our aim is to introduce common design language and offer some interdisciplinary perspective, to support the conversations that designers typically must facilitate in order to organize their work in the field of global health.

While open ended *Discovery* reveals new possibilities grounded in real world observations, synthesizing insights and *Defining* contextual priorities and constraints enables the design team to converge on a way forward. Exploring many aspirational and wild ideas for possible solutions gives *Ideation* an open-ended character, while selecting a few *Prototypes* to build introduces questions of technical practicality and involves focusing on a way forward. *Testing* prototype interventions in practice will again generate new insights and reasons for course-correction, while *Refining* in light of these tests enables designers to iterate towards increasingly worthwhile artifacts. The whole process is characterized by hands-on engagement and iteration – two themes we will discuss further below.

Much like the UK Design Council's well known ‘double diamond’ visual of the design process (Design Council, 2007), Figure 1 portrays activities that generate new possibilities as diverging away from the center line. The alternating activities of synthesis and scope definition, building prototypes and iteratively refining them involve convergence and increasing focus. Alternating between divergent activities that ‘create choices’ and convergent activities that ‘make choices’ safeguards against groupthink and the problem of prematurely converging on a way forward. This entails reexamining what problem really is (or could be) addressed, potentially reframing the problem from different points of view rather than starting with a specific technology or solution already in mind. Since 2002, the designer Damien Newman's design squiggle has been widely used to represent the kind of sensemaking and openness to ambiguity that this entails.^7^ The twists and strange loops of this non-linear approach reflect the fact that insights at any point can lead to major iterations that entail revisiting earlier work. Despite this openness to ambiguity and apparent messiness, the approach is repeatable insofar as there is a disciplined flow and increasing convergence to the process overall. Table 2 summarizes two respects in which designing for health differs from discovery and innovation frameworks that are more prevalent in medicine and public health research.

**Table 2.** How design differs from prevailing approaches in global health.

Design approaches differ from and can complement prevailing approaches to research-based intervention in medicine and public health. This table highlights several tendencies that merit further reflection.		
	Medicine & Public Health	Design
Formative Research	Experts review literature, apply existing health outcomes evidence and behavior change theory. Stakeholders may be consulted in interviews or focus groups (Whittaker, Merry, Dorey, & Maddison, 2012).	Hands-on approach to exploring possible futures. Often involves fieldwork with stakeholders, eliciting input with sketches or prototypes. Theory and health outcomes evidence from other settings may be consulted.
Iteration	A linear, step-wise process in which pilot trials are replicated in increasingly larger and more ordinary clinical settings. Clear evidence of effectiveness is the end point.	An iterative process begins with open-ended discovery. After evidence of effectiveness is established, iterative redesign remains central to service integration and scaling up implementation.

### 3.1. Hands-on engagement versus hands-off approaches

In perhaps the most widely-cited work of design scholarship ever published, Schön (1983) described design as a reflective *conversation with materials*. He observed that the process of making is typically complex, because the designer's moves often have intended and unintended consequences. The direction of a design project emerges in practice, because the designer cannot fully predict or control how the materials will respond to her initial attempts to shape them. Making sense of a situation through hands on engagement can extend into social research activities through which designers explore the perspectives of participants or users. This *discovery* work is often described as ‘fuzzy’ because initially there is high ambiguity about the context and about what form the artifact may take, including whether it may involve software, hardware, new services, etc. Conducting fieldwork and reflecting on the perspectives, tacit situational awareness and everyday practical workarounds of a health system's participants can clarify which interventions are likely to work in a particular setting. Often discussed in terms of cultivating empathy, ethnographic methods in design research can *make work visible* (Suchman, 1995) so that technical possibilities can be re-crafted to integrate more intuitively into existing patterns of technology use and care work. Such insights are vital for international teams hoping to support local maintenance, repair, and ownership of open-source tools, longstanding aims in the appropriate technology for development community (Schumacher, 2011). In this vein, we might attend to locally available phones, ordinary rather than ideal infrastructure, and the working knowledge and emotional responses of local partners with the summary phrase *make it familiar*.

**Figure 2.**
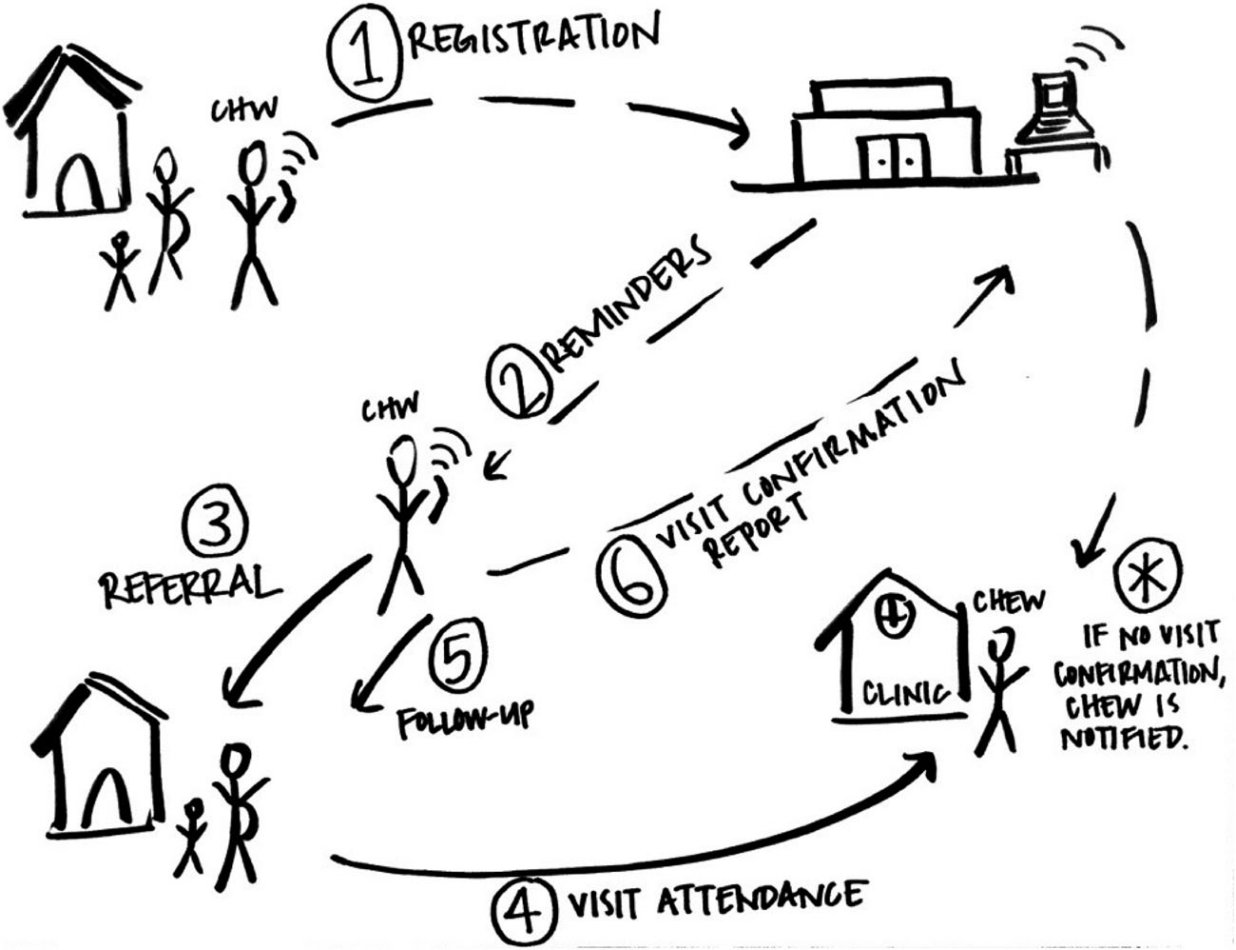
Sketch of an SMS-enabled antenatal care intervention. In this example mockup of the workflow or mechanism of action for an antenatal care (ANC) intervention, (1) a CHW registers a pregnant woman via SMS; (2) software installed at a hospital automatically creates a schedule of appropriately-spaced ANC visits and sends the CHW personalized notifications before each appointment; (3) the CHW re-visits the household to refer the woman for ANC; (4) typically the pregnant woman visits clinic; (5) the CHW follows up a few days later and (6) sends an SMS to confirm that the appointment was attended. If no SMS confirmation is received, the CHW's manager (the CHEW) is automatically notified. Partners often find such workflow sketches more participatory and accessible to input (especially across language, culture, and power barriers) than technical product specifications or detailed written/verbal descriptions alone. New projects involve many variations of such sketches as designs for technology and service delivery co-evolve.

While qualitative interviews, focus groups and even participant-observation are well established in designing digital health interventions (e.g. Whittaker et al., 2012), design approaches contribute a few more hands-on methods to the practitioner's toolkit. Several differences between design research and typical health research methods have to do with the relationship between understanding situations and changing them. In design-oriented research, understanding is always in the service of imagining a better future, which means that objectivity and controlling bias are not the highest aims. Designers elicit people's views with sketches, photographs, interactive role-plays, mockups and prototypes (see Figure 2) that allow people to lay their hands on the future:

Design artifacts such as mock-ups can be most useful in early stages of the design process. They encourage active user involvement, unlike traditional specification documents. For better or worse, they actually help users and designers transcend the borders of reality and imagine the impossible … they encourage “hands-on experience,” hence user involvement beyond the detached reflection that traditional systems descriptions allow. (Ehn & Kyng, 1992)

Watching people work with prototypes can help designers to gauge participant response bias, the well-documented problem of informants telling designers what they think the designer wants to hear (Dell, Vaidyanathan, Medhi, Cutrell, & Thies, 2012). However, the professional designer is not necessarily the primary decision-maker in this role; they might also act as a facilitator and champion of stakeholder priorities. The aim is to make the design process transparent and generative, exploring many variations of multiple contrasting possibilities before ultimately converging on an opportunity for intervention.

Grounding technology design in evidence of people's everyday experiences means that the process is more tailored to the concrete details of particular local situations than is the case in efforts to design interventions based on universal behavior change theories (Michie, van Stralen, & West, 2011) or evidence from health outcomes trials in other settings. As one scientific working group put it, ‘“one size fits all” seems distinctively non human centered’ (Kling & Star, 1998). When human-centered designers work in circumstances that they consider to be unique, they typically take insights from experiments in other settings as inspiration or as guideposts rather than as rules. They proactively document and adapt to local challenges even where those challenges were not observed in other settings or anticipated in project specifications or protocols. The relative emphasis on adapting in light of local evidence over applying generalizable evidence initially surprises many medical practitioners, particularly if they have been trained to use implementation frameworks that emphasize ‘fidelity’ to prior studies (Carroll et al., 2007). Nonetheless, the complexity of design work makes such an approach necessary. The Community Health Toolkit's open-source codebase has been updated more than 10,000 times spanning over 225 scheduled releases^8^ in addition to innumerable paper prototypes and custom code for specific implementations that did not result in changes to the core software framework. The aim is for all of these adaptations to be rooted in data and evidence of people's experiences, yet this complexity means that there are far more variables to consider than there are relevant, controlled experiments on which to base design decisions.

Designing in this sense is a disciplined and reflective practice (Schön, 1983); it is complementary to and should not be conflated with the expert knowledge of experimental science (Cross, 2001). This is not to say that medical scientists and design practitioners can afford to ignore one another, however. As discussed in the ISO standard on human-centered design (2010), the Principles for Digital Development (Waugaman, 2016) and elsewhere (Pagliari, 2007; Roberts, Fisher, Trowbridge, & Bent, 2016) assembling multidisciplinary teams is a valuable opportunity to make such differences generative. That is to say, human-centered design is by definition an explicitly multidisciplinary practice concerned with integrating diverse perspectives. Diverse teams that grasp the relevant health outcomes research, qualitative insights, relevant behavioral theories, and how these perspectives can feed into a cooperative design process are generally viewed by designers as more capable of innovative thinking. Diverse teams may reframe a problem from more points of view, or attend more deeply to local complexities before proceeding to wider implementation or evaluation. In contrast, taking project scope and problem definition for granted can result in decisions that are biased by the assumptions or limited personal experience of funders and implementers.

### 3.2. Iterative prototyping versus linear, stepwise approaches

Sketching and hands-on activities that provoke open-ended feedback can yield surprising insights and uncover hidden complexities and unintended consequences that would have been inaccessible in surveys or structured interviews. Addressing the whole stakeholder experience means that designers often evoke feedback on matters that initially seemed tangential. In our experience, common cases include revealing that an intervention for one health issue cannot proceed without changes in adjacent programs, or a practical manner in which local electricity access or the need to coordinate with other health workers can constrain technology use. Working in this way yields insights about the practicality, perceived value and potential adoption of an intervention before initiating a costly pilot study. It has the benefit of avoiding health worker frustration or burnout with interventions that should have been more thoroughly vetted before being implemented. By reframing "human error" or "poor adoption" as mandates to redesign digital tools or service workflows, iterative approaches also relieve users of the expectation to embrace pre-determined interventions that might not have reflected their priorities. This tendency to reframe human error, and the common expression *the user is never wrong,* grow from the fact that systems thinking has long permeated the design community (Bannon & Ehn, 2013).

Iterative prototyping typically continues through design-in-use, reconfiguring technologies in response to difficulties that only emerge through implementation and sustained use of technology. Designers cannot always predict or control such complexities but they can adapt to them when they emerge in practice. Some researchers refer to this iterative integration of proactive design and organic evolution as *guided emergence* (Holeman & Barrett, 2017; Sein et al., 2011). A consequence of taking emergence seriously is that technology design cannot be fully divorced from the redesign of work practices and the organization of health systems. In systems design, terms such as sociotechnical (Scacchi, 2004) and sociomaterial (Holeman & Barrett, 2017) underscore that technical, social, biological and ecological systems are entangled in practice and should be considered holistically in design projects.

A mindset and practice of iteration permeates, but is not unique to design thinking. It is similarly central for example in agile software development methods (Beck et al., 2001). Nonetheless, iteration bears special reflection for designers in global health because widely accepted models of medical evidence stipulate intervention design, evidence generation, and replication as separate phases (Tomlinson et al., 2013). An iterative design practice necessarily blurs the traditional separation of design and implementation, revisiting key design considerations at iteration meetings throughout every stage of discovery, scale up and integration of services, and broader health systems strengthening. This iterative integration of design within the wider human context of implementation is precisely how a design approach is thought to be amenable to solving complex or wicked social problems (Rittel & Webber, 1973; Schön, 1983).

It is important to recognize that applying design thinking to complex or wicked social problems (Rittel & Webber, 1973; Schön, 1983) is conceptually and practically distinct from efforts to cultivate a more human-centered design practice. Every aspect of the design process can be more confined to the expert designer and her materials, or more grounded in people and their priorities. For example when Peter Rowe wrote the book *Design Thinking* in 1987, the conversation about stakeholder participation (Simonsen & Robertson, 2012) and grounding key design decisions in evidence of other people's experiences (Norman, 2016), was far less prominent than it is today. It remains the case that not all design professionals appreciate the term ‘human-centered’ or the practices it implies – for some designers hands-on engagement will mean a trip to the studio or time alone with a sketchbook, rather than fieldwork with end users. This spectrum of more or less human-centered aims and practices in the design community can make it difficult for outsiders to grasp what distinguishes human-centered approaches and how they might complement the more general practice of design for health. To make this spectrum more visible, we offer a description of human-centered design, and then survey three themes that often characterize human-centered approaches.

## 4. What can human-centered approaches add to design for health?

### 4.1. Human-centered design as an umbrella term

Human-centered design is a flexible, yet disciplined and repeatable approach to innovation that puts people at the center of activity. As an umbrella term, it speaks to practices for prioritizing people's aspirations and ordinary experiences when imagining and implementing complex systems, services, or products. According to the International Standards Organization (ISO, 2010), human-centered design is a complex practice characterized by six principles:

- the design is based upon an explicit understanding of users, tasks and environments;
- users are involved throughout design and development;
- the design is driven and refined by user-centered evaluation;
- the process is iterative;
- the design addresses the whole user experience, including the context in which the user finds his/herself;
- the design team includes multidisciplinary skills and perspectives.

The ISO's technical definition is a helpful starting point, yet it has long been recognized that there is no universally-agreed view of human-centered design (Kling & Star, 1998). Taking a historical perspective, Bannon (2011) emphasizes that human-centered design research and practice have evolved thanks to influences from human factors and human–computer interaction, the methodological contributions of anthropologists and sociologists, organizational information systems research, user-centered design, participatory design, and the more craft-oriented design professions. Design efforts focused on social innovation (Brown & Wyatt, 2010), international development (Brand & Schwittay, 2006; Dearden & Rizvi, 2008; Oosterlaken, 2009) and global health (Holeman et al., 2014; Holeman, Cookson, & Pagliari, 2016) continue to push this multidisciplinary practice in new directions.

Given the obvious and important differences among these approaches, proponents and critics of human-centered design sometimes associate the term narrowly with just one tradition. For example, early work on user-centered design was closely associated with experimental psychology – it is telling that the first edition of Donald Norman's classic book *The Design of Everyday Things* was titled *The Psychology of Everyday Things*. In contrast, Computer Supported Cooperative Work (CSCW) researchers have often favored ethnographic perspectives. The ISO standard on human-centered design (2010, p. 2) observes that many regard user-centered design as a synonym, yet those who see human-centered design as encompassing the CSCW tradition of design ethnography often disagree (Bannon, 2011). In another telling example, Brown and Wyatt (2010) discuss human-centered design and design thinking interchangeably. However, historically design thinking and user-centered design did not emphasize stakeholder involvement in the manner that characterizes participatory co-design (Brubaker, Jensen, Silungwe, Sheppard, & Yang, 2017). Those who see human-centered design as reflecting the influence of participatory approaches (Bjögvinsson et al., 2012; Kling & Star, 1998) *and* a scientific approach to user research (Norman, 2016) will tend to see human-centered design as a more encompassing umbrella term. Alongside these developments in design research, the branding and promotion of these terms in industry and the popular media undoubtedly have also shaped how they are used in practice.

For practitioners, it has become the norm to deliberately switch among these kindred approaches in order to better grasp the distinctive challenges of particular design cases. This does not necessarily require any attempt to systematically unify or homogenize these traditions into one singular conceptual definition for human-centered design. Rather, in practice it can be justified on the grounds that they bear a certain family resemblance. The theories, methods, mindsets, and values that fall under the human-centered umbrella all emphasize a more holistic attitude toward the human person, including the social and cooperative dimensions of their humanity. Taken together, this toolkit of approaches underscores the wholeness of life that people inevitably bring to each day in the workplace or attempt to interact with artifacts. It is this broader turn in the design community that we might call the human-centered paradigm shift (Bannon, 2011).

While using human-centered design as an umbrella term can support a lively pluralism, it also means that there is no precise consensus, among practitioners or researchers, about what the 'human' part of the term is supposed to mean. Practitioners often use these terms casually to invoke any design project that takes people or social issues seriously. This strikes many others as problematic, in light of recent critiques of buzzwords and empty rhetoric:

Empowerment and agency and human-centricity have come to seem like euphemistic ways to get donors to feel like they are not engaging in neo-colonial practices by defining and determining the presence of healthcare for populations worlds away from their own. (Guardian, 2015)

While hollow design rhetoric recently has surfaced in a distinctive way, the problem is hardly new. Two decades ago, a scientific working group wrote that they ‘were especially concerned that the term ‘human-centered’ could easily become a trivialized buzzword that could casually be slapped as a label onto any computer application that seemed to help people’ (Kling & Star, 1998). In their view and in a great deal of subsequent work, human-centered design involves specific conceptual, and even ethical commitments related to stakeholder participation, supporting skilled work, and surfacing the values at stake in design projects.

### 4.2. Participatory design

Participation is a touchstone for those who associate the term human-centered with engaging stakeholders as partners, rather than viewing designers as experts and potential users as mere informants. For many in the international development community, longstanding traditions of participatory development (Chambers, 1994; Dearden & Rizvi, 2008; Freire & Freire, 2004) are a relevant point of reference. In popular media there is a common perception that human-centered design was developed by private sector innovators (Lee, 2015a), but participatory design actually emerged in the 1970s out of Scandinavia's workplace democracy movement (Simonsen & Robertson, 2012). In partnerships among academics and labor unions, participatory design was explicitly political (the democratic view that workers should have a say) and explicitly pragmatic (people are more likely to adopt tools that reflect their priorities).

Many now use the term co-design together with participatory design (Sanders & Stappers, 2008). Some work emphasizes engaging users early in the process, as partners in idea generation rather than as passive informants whose role is to provide feedback on concepts developed by expert designers (Yoo, Huldtgren, Woelfer, Hendry, & Friedman, 2013). This is not to say that human-centered design is inevitably democratic; participation can be neglected or depoliticized in a manner that grants no real power to non-experts. For this reason, if we are to claim that a design process was human-centered because it ‘involved the user,’ it is worth documenting whether or how stakeholder participation substantively shaped the outcomes of the design process.

### 4.3. Supporting human skills and cooperative activity

For many the term human-centered also evokes a commitment to supporting skilled work, or even augmenting people's skills and competencies. In the classic essay *From Human Factors to Human Actors*, Bannon (1992) opens with a telling quote:

Man is one of the best general-purpose computers available and if one designs for man as a moron, one ends up with a system that requires a genius to maintain it. Thus we are not suggesting that we take man out of the system, but we are suggesting that he be properly employed in terms of both his abilities and limitations.

This perspective is perhaps best understood in contrast to an earlier approach to workplace automation, in which technology was often used to improve efficiency or productivity by making workers obsolete or monitoring and controlling their behavior. Since the 1940s, proponents of sociotechnical design have decried industrial technology projects that they described as undercutting skilled work or ‘deskilling,’ reducing worker autonomy, increasing monotony, and generally having ‘dehumanizing’ effects on the workplace experience. Having documented how technology-driven efficiency can come at the expense of human dignity, such critiques emphasize the importance of “designing human systems” (Mumford, 1987) in an iterative and participatory manner.

As the reach of automation extended out from the factory and into the office, efforts to computerize were often met with a frustrated, anti-computerization sentiment. Reflection on these anti-tech social movements led to calls for ‘learning how to humanely integrate new computer-based technologies into routine social life’ and the search for ‘a coherent alternative humanistic vision for appropriate computerization’ (Kling & Iacono, 1988, pp. 226–236). Early discussions of human-centered design pleaded that we design new information systems without repeating the dehumanizing mistakes of technology design in the industrial revolution (Cooley, 2000). Subsequent work has affirmed that these calls for a more humane approach to automation and computerization were influential precursors to human-centered design as we know it today (Bannon, 2011). Thus by the time the term *human-centered design* was gaining currency in the 1990s, researchers were already drawing on decades of studies to argue that ‘human-centered systems are designed to complement humans’ skills … design predicated on merely replacing or automating human activity is not human centered’ (Kling & Star, 1998, p. 24).

### 4.4. Human values in context

Another aspect of human-centered design has to do with recognizing the human values at stake and the moral consequences of particular design projects. In addition to the priorities intentionally supported by the systems being designed (e.g. averting child mortality), it is important to explore stakeholder values and any value conflicts that may emerge in practice. This premise is based on decades of research documenting unintended consequences and *the dark side of information systems* efforts gone awry in diverse social and organizational contexts (Kling & Star, 1998). When there is sufficient openness to acknowledging the conflicts that often emerge in the wider human context of implementation, proponents of human-centered design generally hold that many of these issues can be dealt with through pragmatic redesign efforts.

The growing literature on value-sensitive design (Friedman, 1996), has developed theory and formal methods for technical and empirical investigations at the individual, group, and societal levels of analysis. An important conversation here has to do with the merit of listing and implementing universal human values (honesty, reliability, transparency, etc.), relative to emphasizing responsiveness to the values of the specific people likely to be affected by a particular intervention (Borning & Muller, 2012; Houston et al., 2016). Even when human-centered designers do not embrace the formal methods of value-sensitive design, we would do well to reflect on the moral stances we imply when we talk of ‘human’ concerns and use related words to describe our work.

For many designers, working in the fields of global health and development has meant reflecting on matters of inequality and health equity. This is not a recent development; in his 1972 classic *Design for the Real World*, Victor Papanek drew attention to ‘the basic survival problems of humanity today’ and argued forcefully that ‘in a world brought nearly to its knees by abject want, a preoccupation with “making things pretty” is a crime against humanity’ (Papanek & Fuller, 1972, p. 327). Working in such contexts, some designers discuss human-centered perspectives alongside human rights, human dignity or humanitarian concern (Buchanan, 2001). When designers use expressions such as human-centered, humanitarian, humanity, humane, (de)humanizing, human dignity, and human rights in this manner, they are not all working from the same definition. Their language is not united by a single overarching characteristic, and yet we can clearly recognize a cluster of overlapping meanings with a discernible thrust. In practice, such language implies values or moral stances that can and often do shape the course of design work. In summary, substantively human-centered design efforts often involve:

- meaningful and documented participation of people who will use new systems in their routine activities or otherwise be affected by them;
- supporting cooperative activity and augmenting people's skills, rather than using technology primarily for purposes of efficiency or managerial control; and
- concern for the whole person and their life experiences, reframing purely technical issues in relation to people's values and the broader human context of implementation.

Some critics describe human-centered design as a method for solving narrowly technical problems (Janzer & Weinstein, 2014), and this is understandable given that many practitioners now invoke the feel-good expression ‘human-centered’ to describe whatever manner of design practice they find convenient. Yet our synthesis of research on human-centered design clearly suggests a more substantive and challenging remit. Some design projects will reasonably emphasize one of these themes while leaving others in the background. However, invoking the rhetoric of human-centered design while working in a way that directly undercuts any of these themes is at best poorly informed, and may be hypocritical. In the following section we illustrate how these conceptual issues can be relevant to practice, by offering reflections on our own work as designers.

## 5. Reflections on human-centered design at Medic Mobile

### 5.1. Situating Medic Mobile’s design practice

The practical specifics of our design work have evolved considerably as our software toolkit has gained new functionality, and our team and open source community have grown. Medic Mobile's early projects focused on interactive, conversational text messaging for care coordination. Through these projects we accumulated deep familiarity with CHW workflows and communication needs, the robustness of SMS in occasionally connected settings, the real limits of 160 characters and nine-button keypads, and myriad operational challenges related to CHW training, maintaining basic phones, and replenishing airtime and electricity.

The focus of our design efforts expanded in 2011 when we began supporting task management and reminders via SMS. To gather enough patient information to generate personalized care schedules, we designed the first SIM card applications for health (Holeman, Yembrick, et al., 2016), as well as what we call Text Forms, a structured format through which CHWs can submit data such as patient registrations via text message. More dramatic changes in user experiences and the scope of our design efforts came in 2014, when we began using smartphones to provide decision support, longitudinal health records, and richer task management interfaces. These data-intensive features engendered partner requests for more analysis tools for CHWs and the teams that support them. Now that we have designed analytics dashboards for community health workers, their supervisors and for health systems managers, understanding key metrics and performance management workflows for each new implementing partner is more central to our design practice than was the case before our software supported these features.

We now design for and with community members, CHW supervisors, nurses, and managers, in many languages, for a range of phones, tablets and computers, in hard-to-reach settings with or without connectivity. Most of our projects remain focused on care provider workflows, which are complex and remarkably diverse from place to place. Each time we redesign our tools to address a new health issue, we must immerse ourselves in the relevant science, engage policy makers and undertake fieldwork to document the nuances of health worker behavior. We have supported antenatal care, postnatal care and family planning, child health including immunization and nutrition services, early child development, outbreak surveillance, cervical cancer screening, and HIV and TB services, often with great care for how workflows are integrated across programs over time. A growing number of our partners have documented remarkable health outcomes improvements through an approach to health system strengthening that integrates services across disease-centric programs and levels of the health system (Bonds & Rich, 2018). We now take part in a coalition that advocates for scientific and policy bodies to rethink the design of community health systems with respect to e.g. routine training and supervision, removal of user fees, salaries, and data feedback loops (Ballard et al., 2017). These experiences in integrated health systems strengthening have underscored the unavoidable relevance of a point made earlier – benefiting from digital health often entails an integrated redesign of new technologies and the health system as a whole. Digital tools are of little value to health systems that remain in general disarray.

With over 25,000 people using this software to support nearly a million discrete health services per month, quantitative usage statistics have come to play an increasingly important role in our design practice. Routine reviews of granular system usage data often reveal puzzling trends that we can only understand through targeted fieldwork. At the same time, when our fieldwork reveals rich qualitative insights about health worker practices, this often leads to targeted analysis of usage data that can help us understand the prevalence or consequences of the practice. In this way our longstanding practice of designing with end users, our routine analysis of system usage data, and our more recent data science efforts are becoming intertwined. This is common among design teams at tech companies; we see it as a promising opportunity for global health.

Our software also supports a range of rapid diagnostic tests, sensors such as the SimPrints finger-print scanner (Storisteanu, Norman, Grigore, & Norman, 2015), and integration with complementary health information systems such as OpenMRS and DHIS2 (Braa et al., 2004). Through a partnership with Nexleaf Analytics, we helped to design an internet-of-things device that remotely monitors the temperatures of vaccine fridges (Holeman & Barrett, 2017). Many of our projects involve close attention to paper information systems such as referral slips and patient health booklets, which remain ubiquitous in the places we work. Concretely reshaping these complementary tools is often beyond the scope of a particular design project. Nonetheless, complementary tools feature prominently in our exploration of particular local health systems and in key design decisions, because we are aware that our software can integrate more or less artfully with this wider technical ecosystem.

Overall, our toolkit of materials, strategies, and models of healthcare delivery has evolved considerably. We have summarized these concrete particulars of Medic Mobile's design practice in order to emphasize two insights which were relatively more implicit in the conceptual review of human-centered design above. First, like any serious design team, our expertise lies not only in abstract generic design principles or a human-centered mindset, but also in our deep intuitive familiarity with a distinctive repertoire of materials and practices. Having taken an online course or even studied at a good design school is no substitute for having had conversations with hundreds of community health workers, or for familiarity with the technical features of a software ecosystem that will shape a given design project. Second, this diverse range of design experiences informs our view that human-centered design is a flexible and repeatable approach, relevant to a wide range of settings and health interventions. While our experiences are limited in some respects, we have benefited from human-centered design in a sufficient range of innovation projects to reasonably expect it to prove useful in additional settings.

In the following paragraphs we aim to flesh out more of the concerns that we reviewed above: iteration, hands-on participation, human skills, and human values. We do so by reflecting on the specific ways we have found them challenging and helpful in our own work. Finally, we reflect more broadly on the difficulties of growing a design team, and on the prospect of practicing human-centered design as a pragmatic way of accompanying health workers in their struggle for health equity.

### 5.2. Making sense of stakeholders through personas and iteration

In 2010 Medic Mobile began a partnership with the Kenyan Ministry of Health and the NGO Kilifi Kids. With a focus on antenatal care (ANC) and immunization, we involved nurses and community health workers in designing and testing an evolving toolkit of technologies and supported workflows to improve coordination, access, and quality of services. After several rounds of fieldwork and interviews, we converged on a plan to support the workflow outlined above in Figure 2.

Our design process involved creating personas for key users of the proposed system. A persona is a generic character that tells the story of a larger group of users, including typical demographic information, skills, activities, and priorities. In this project ‘CHW Janet’ would use a basic mobile phone and send SMS messages to register pregnant women and children in her community. Our initial design for a data dashboard revolved around a ‘Nurse Mary’ persona, because she was responsible for clinical aspects of ANC and immunization and because during initial design sessions, nurses seemed interested in using patient data to support follow up.

Several months after initial implementation, we conducted another round of fieldwork and discovered some local workarounds that had not been apparent when we monitored system data remotely. After training, public health oriented CHW supervisors (rather than nurses) had taken responsibility for the fact that not all CHWs were submitting data as expected. As a result these supervisors had become a key audience for data generated by this system, but they were using a dashboard that had been designed for the more clinical Nurse Mary role. While CHW supervisors had participated in early design sessions, it had proven impossible for any of us to imagine the specific way that they would come to use the new system until it had been implemented and in use for a while.

This observation proved to be a breakthrough in the evolving design process. Our initial framing, focused on integrating community and clinical aspects of ANC and immunization, was not necessarily wrong. Yet a more fruitful opportunity emerged through ongoing design-in-use. While documenting key personas can help build empathy and concrete evidence of stakeholder experiences into the design process, making sense of which personas to focus on cannot be taken for granted. Our lesson in this project had as much to do with iteration in general as it did with personas in particular. Engaging health workers as participants in the design process is not a ‘once and done’ task to be associated only with formative research, but an ongoing opportunity with each new design iteration.

### 5.3. Hands on design research with sketching and participatory design cards

While we have experimented with many strategies for eliciting stakeholder experiences, sketching remains one of our more reliable and favored methods (see Figure 2 for an example sketch). It is flexible enough to facilitate many aspects of the design process, and it is more accessible and participatory than detailed written ethnographies or technical product blueprints alone. Sketching as a visual note-taking strategy enables interviewees to validate the information we capture as we are capturing it, and to offer real time feedback in the form of general enthusiasm or skepticism, body language or detailed comments. Particularly when working through translators, it is easy to misinterpret what someone has said or to miss important points, and sketches provide a degree of transparency that is difficult to achieve with written notes.

Sketching in real time requires specific skills, though. Synthesizing large amounts of information on the spot, incorporating new insights, or making live corrections takes practice and creative confidence. In 2012 we began experimenting with ways to encourage health workers and our own design staff to sketch more throughout the design process. Many of the community health workers we support have not used whiteboards before; we should not let this prevent them from articulating meaningful insights about communities where they are the local experts. We developed *design cards* as a way to break down the most common elements in health system workflows so that our team and partners could build sketches quickly and dynamically (Principles for Digital Development, 2017).

**Figure 3.**
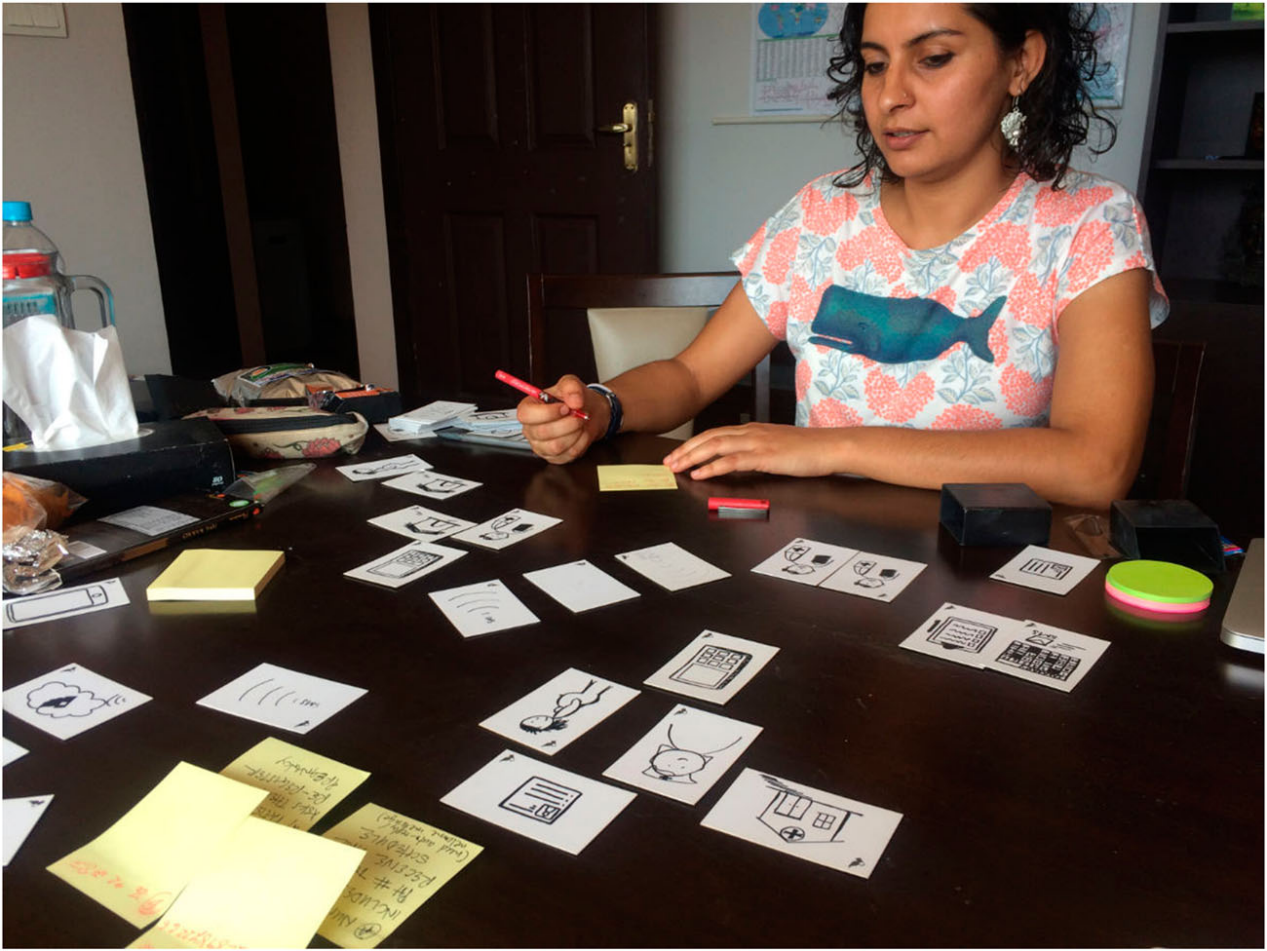
A Medic Mobile designer in Nepal using participatory design cards.

We have used these cards for several years now because they often have an inclusive, participatory effect for stakeholders ranging from community health workers to scientists and policy makers. Particularly when working across language barriers, design cards take some reliance off of the translator and refocus the conversation on the system being laid out on the table. This is important not only for North American and European staff, but also for example when Kenyan designers are working in a part of Malawi or Uganda that does not speak Swahili. Even when collaborating in the same language, design cards give both parties another hands-on tool with which to communicate their ideas and explore scenarios in a more tangible form. We point to or re-arrange the cards as we speak and participants often do the same; this helps to create a more direct relationship among us and a feeling that we are creating something together. This experience highlights that substantive participation is not the result of mere intentions; it is an accomplishment that can benefit from certain design skills (Figure 3).

### 5.4. Super powering health workers versus ‘just telling and selling’

From 2008 to 2010, Medic Mobile's efforts in Malawi demonstrated that interactive text messaging could be used to coordinate care and strengthen community health systems in settings of poverty (Mahmud et al., 2010). However, as our toolkit spread, a growing number of potential partners began to ask about using it to blast health information directly to household phones. For many, the apparent promise of these technologies was the possibility of making community health workers obsolete; they cited the expense and complexity of CHW programs and imagined automating away human error. From our perspective, they seemed to associate the promise of technology with disrupting or replacing health systems, rather than strengthening them.

For most people who are excited about technology-driven efficiencies, the awareness that human workers might be replaced is relatively more implicit. The deskilling tendency of technology projects is certainly widespread though; some research suggests that digital interventions emphasizing passive reception of messages and a focus on individual behavior change have seen far wider scale than the use of interactive technology to strengthen health systems. Noting the relative lack of systems that people can interact with in a self-directed manner, one review of digital media in public health characterized the majority of work as ‘just telling and selling’ (Clar et al., 2014).

Human-centered design does not offer fixed rules for how best to support skilled work, but it does establish a process for reframing design challenges and an expectation that we take the human consequences of innovation seriously. A design challenge such as "what kinds of SMS message content are engaging enough to change poor people's health seeking behavior?" might reflect a preconception that the human touch of a caring health worker is non-essential. In contrast, the design challenge, "how can we better support health workers in the task of coordinating care and mobilizing communities?" reflects different preconceptions, and some would say more human-centered aims. Framing community health workers as an undesirable burden on health systems would not necessarily be inconsistent with the application of design thinking or user-centered design, if we conceive of the patient as the user. For this reason, the human-centered emphasis on people's skills in the wider implementation context has seemed particularly relevant in our work. In our experience, technology can be designed to augment human skills as easily as it can be designed to deskill and automate. As Toyama (2015) puts it, technology amplifies underlying human forces. We like to think of our work as building tools to give health workers *super powers*, rather than building tools that take humans out of the loop.

### 5.5. Human-centered design as accompaniment

The language of equity and humanity has long been important in the global health community. As Lancet editor Richard Horton put it, ‘global health is an attitude. It is a way of looking at the world. It is about the universal nature of our human predicament. It is a statement about our commitment to health as a fundamental quality of liberty and equity’ (Farmer, Kim, Kleinman, & Basilico, 2013, p. xv). Given their close contact with patients, many health workers recognize that the poorest people are systematically more likely to be exposed to disease and harm and less likely to access working health systems or robust technologies (Farmer, Nizeye, Stulac, & Keshavjee, 2006), let alone opportunities to redesign either.

Responding to such inequities, Farmer and Gutierrez argue that we assert our humanity in the struggle for a more just and caring society, in which people can live with dignity and become the authors of their own destinies (2013, p. 57). Their call to build ‘a preferential option for the poor’ is grounded in an ethic of partnership and solidarity with people whose priorities and humanity are too often neglected by society at large. This language and moral outlook suggest a broader and more challenging remit for human-centeredness. In an interview with Wired magazine, Farmer was asked to address how his perspective on human-centered design might work in practice:

In Haiti I would see people sleeping outside the hospital with their donkey saddle under their neck – they’d been waiting there for days. And no one was asking them, “What are you eating while you’re waiting? What is your family eating while you’re gone?” We have to design a health delivery system by actually talking to people and asking, “What would make this service better for you?” (Roper, 2013)

To rephrase this challenge in more general terms, designers who build technologies or solve technical problems can hardly claim that their work is human-centered if they systematically ignore human rights or humanitarian concerns that are part of daily life for the people they purport to ‘design with’ or serve. Attending more broadly to human experience in this way involves looking beyond the design of discrete technologies to reimagine services, the organization of health systems, and broader social arrangements that pattern who receives equitable care and who does not. This wider scope for design is probably inevitable, at least when working in settings of poverty, if we take seriously a contrast that distinguishes human-centered approaches from a focus on ‘the user’ in the first place – the call to reframe local matters of usability or task completion in relation to the broader lived experiences of concerned persons. Some will see this as hubris, a presumption that savvy methods and attitudes will enable the designer to resolve all conceivable difficulties. But it can also be seen as a call for humility; the difference depends on how we designers locate ourselves relative to our partners and take responsibility for recognizing even those complexities which we lack the power or imagination to resolve.^9^

To be sure, designing in a manner that addresses practical problems today, without sidelining more systemic inequities, is no easy feat. In this regard, the Medic Mobile design team has long taken inspiration from the *pragmatic solidarity* of Farmer and his colleagues at the non-profit organization Partners in Health. In Partners in Health's approach and in other institutions around the world, community health workers are called *accompagnateurs*. The name implies that their central remit is not only to deliver efficient biomedical services, but to accompany patients, to ease suffering and to offer their caring presence as an antidote to despair. As Farmer puts it, accompaniment ‘means just what you'd imagine, and more. To accompany someone is to go somewhere with him or her, to break bread together, to be present on a journey with a beginning and an end’ (Farmer, 2013, p. 234). While the term accompaniment is somewhat elastic, it is also clearly different than a paid consultancy, a one-off project or a short-term visit. Accompaniment typically implies staying the course until the person or people being accompanied consider the journey completed.

As designers with niche expertise in digital technology, accompanying a community in their struggle for health equity affords us an active role in an ongoing process of social change. It highlights the quality of our relationships with community members, without taking for granted that everyone will share fully in our motives and optimism, or be able participate on equal footing. Accompaniment also implies prioritizing design outcomes that matter to our partners, without overstating the role that we, or our technology are likely to play in their broader agendas. This vision of accompaniment emerged in Latin America as a feature of liberation theology, and it has been influential in the global health community (Farmer & Gutiérrez, 2013). It underscores that building a preferential option for oppressed people necessarily involves building it with them; taking part in their liberation involves standing by their side and sharing their path for a while. This perspective shares important themes with other social justice, feminist, and postcolonial approaches to design (Bardzell & Bardzell, 2011; Costanza-Chock, 2018; Escobar, 2018; Irani, Vertesi, Dourish, Philip, & Grinter, 2010; Janzer & Weinstein, 2014; Staton, Kramer, Gordon, & Valdez, 2016).

In the global health and development community, where key design decisions typically are made by experts who are geographically and socially far removed from people who may live or die by their judgment, the notion of accompaniment could not be more pragmatic, or more challenging. In our own work, this commitment is perhaps most visible in how we approach human resources for human-centered design. Medic Mobile now employs fourteen designers globally, twelve of whom are women; the team includes seven Kenyans, four Americans, and one each from Nepal, Uganda, and Senegal. When we look to hire designers, our key priorities include availability for frequent fieldwork and an aptitude for accompanying patients, community health workers, and implementing partners in the design of digital tools. Curiosity, humility, and the ability to put people at ease play major roles in accompaniment. Hiring in this way affords a closeness with our users, not only in geographic proximity but also in the cultural nexus of our design work, in linguistic and social skills that take years to develop. It is not uncommon for our designers and other staff at Medic Mobile to count extended family members among the health workers and communities we serve.

This approach is obviously different than placing most key design decisions in the hands of scientists, engineers, and other experts from wealthy countries. Such attention to local capacity, not just for the use of information systems but for design and innovation, can support generative conversations about what should be designed locally and what is best designed by a global software community (Brown & Nielsen, 2017; Li & Nielsen, 2019). Yet it would be misguided to imply that designers who live in cities like Nairobi and Kathmandu always have the time or travel budget to be as available as they would like for remote health workers. It cannot be taken for granted that the challenges facing poor and hard-to-reach communities will by default be intuitively understandable to urban middle-class designers. And it is not easy for designers to cultivate the skills needed to document design insights in a rigorous and compelling enough manner to shape a design process with many concerned stakeholders and inevitable power dynamics (Bratteteig & Wagner, 2014).

More of our designers have backgrounds in nursing, public health, or project management than have degree-level training in any design discipline, as will likely be the case for other organizations that hire in countries where university-level design programs are less common. This diversity of perspective has advantages, but it also requires intensive investment in skill building. Put simply, it is no easy feat to scale up global capacity for the kind of human-centered design practice that we have described in this paper.

Our experience suggests that it is possible if funding and senior staffing for design are organizational priorities, and if talented junior designers have access to relevant coaching and on-the-job learning resources. Without opportunities for ongoing apprenticeship with more experienced practitioners, our experience suggests that the lived reality of human-centered design can easily fall short of its promise. This has certainly been our experience with design projects that were underfunded, or forced into unreasonably short timelines, or in which senior designers were unable to guide the design process and be as fully present as junior designers and health workers deserved. In this sense a philosophy of accompaniment, both intellectual and practical, may be as relevant for champions of the human-centered design field as it is for the designer who accompanies health workers or the health worker who accompanies her neighbor.

## 6. Implications for research and practice

### 6.1. Implications for practitioners

Human-centered design is an umbrella term with a complex history. While human-centered design only emerged as a recognizable area of work in the 1990s, we argued that it grew out of a wider tradition of design research and practice. Applied to global health, designing involves hands-on engagement and is oriented to evidence concerning the particular situation at hand, though designers often incorporate insights from behavior change theories or evidence from health outcomes trials. And while design begins with formative research, it is never really finished. Design work has a role to play in ongoing iterative cycles of implementation and redesign as interventions are scaled-up into well-integrated, sustainable health systems. Insofar as we care about the complexity and wicked problems that have limited the capacity for digital health interventions to improve health outcomes or health equity at scale, these themes are directly relevant to practice.

The concern that human-centered design has become a buzzword is worth taking seriously. Reviewing classic themes in design research, our paper outlines three ways of judging the substance of rhetoric about human-centeredness in terms of the concrete aims and practices of particular design projects. Participatory methods, the challenge of augmenting rather than replacing people's skills, and attention to human values throughout the course of implementation make human-centered design distinct from other approaches to innovation. A human-centered perspective is more holistic than technology-centered or user-centered perspectives; it encourages us to look beyond technology solutions to more systemic challenges and opportunities. For Medic Mobile and the Community Health Toolkit's growing open source community, human-centered design is an act of solidarity and partnership, a pragmatic approach to accompanying health workers and communities.

This perspective is particularly relevant for projects of global health equity. It has practical implications for how we select where to undertake design projects, who to involve as design partners, and which health issues to frame as design challenges. However, as ICT for development researchers have observed (Anokwa et al., 2009), decisions that prioritize or neglect marginalized communities or diseases that disproportionately affect the poorest of the poor are often made implicitly, before any application of formal design methods. This underscores the difficulty of putting principles into practice, which some practitioner communities have already recognized (Waugaman, 2016). To be sure, there is nothing inevitably participatory or humane in claiming to practice human-centered design, much as there is nothing inevitably caring about the way we provide health care. An ongoing challenge for practitioners and researchers alike will be to explore and document concrete limitations, pitfalls, and success stories in practicing human-centered design for global health equity.

### 6.2. Implications for researchers

In a recent review of ICT for development research, Walsham (2017) highlighted two challenges for future studies: navigating the multidisciplinary nature of the field and the difficulty of influencing policy and practice. In our review of the literature on human-centered design and its relevance to global health equity, these two challenges emerged as clearly interrelated. The academic design literature is so vast and jumbled that when practitioners, policy makers, or researchers in adjacent fields seek an accessible introduction, they are faced with the impossible task of navigating competing schools of thought and making sense of the whole universe of design scholarship before they can explore its relevance to their own work. Understandably, they often end up relying on popular guides that make little effort to cite sources or observe basic standards of scholarly rigor. Practitioners are hardly to blame for the resulting lack of clarity. ICT for development researchers and others who study design and the social good have been too content to publish in insular disciplinary silos and neglect the practical dissemination activities that could raise awareness of our work (Harris, 2015).

The implication for researchers is clear: it behooves us to offer broad perspective on human-centered design, taking the contributions of multiple design fields and the pragmatic concerns of practitioners seriously, as we have tried to do here. It is up to us, not only to practitioners and policy makers, to determine whether the remarkable surge of popular interest in human-centered design will devolve into buzzwords and empty rhetoric, or coalesce in support of more substantive and humane approaches to innovation. Rising to meet this challenge will entail more than producing rigorous publications. As we suggested in the introduction to this paper, it will entail linking these resources with *networks of action* in which practitioners can learn from one another through ongoing cooperation. With this broader challenge in mind, we will highlight two specific implications of our paper for pragmatic, interdisciplinary research.

First, global health researchers are making great strides in measuring the impacts of digital health interventions on health outcomes (e.g. Lester et al., 2010; Zurovac et al., 2011). Many ICT4D and human-centered design researchers are interested in such health outcomes studies, but it has not always been particularly clear how to relate them to the aims of development or human-centered design. Through a focus on health equity, our article offers conceptual linkages between health outcomes research, perspectives on human development, and the attention to human values that has long animated human-centered design research. In this way, we answer Walsham’s (2017) call for ICT4D researchers to make clear what they mean by ‘development,’ or more broadly the aim of making a better world with ICTs (Walsham, 2012). Given the clear growth of practitioner activity related to human-centered design for global health equity, is our hope that this will grow as an area of research in the coming years.

Second, much of the interest in the poor scalability or reproducibility of digital health interventions in lower-income settings has come from the medical literature (Shuchman, 2014; Tomlinson et al., 2013) and is linked to the growing field of global health implementation research or implementation science (Kim et al., 2013; Kruk et al., 2016). This research community has made important steps towards documenting the ‘real world’ contextual factors that are overlooked or poorly captured by the randomized trials of classic biomedical research. However, this literature often treats design and implementation, and thus design research and implementation research, as separate phases. As a result, some guides to implementation research suggest focusing on ‘implementation strategies’ for spreading existing interventions (Peters, Tran, & Adam, 2013), rather than fundamental, iterative redesign of those interventions.

This artificial separation is significant from a design perspective. Growing awareness of the importance of iterative methods that integrate implementation and ongoing redesign stems directly from attention to the complexities and wicked problems that characterize the broader human context of implementation (Buchanan, 1992; Rittel & Webber, 1973; Schön, 1983). It is also a pragmatic roadblock in places where patients with interrelated health issues (e.g. HIV and TB) must navigate a range of confusingly disjointed programs. Calls for more integrated care can only be meaningfully addressed if interventions, which initially were tested in pilots that focused on a single disease, can be fundamentally redesigned in and through the course of implementation.

The tendency to separate design and implementation is understandable, given that design research is seldom discussed as one of the disciplines that informs implementation research. It is our hope that this paper will serve as a starting point for researchers who are interested in exploring human-centered design, not only as a practical approach to addressing implementation challenges, but as a body of concepts and research methods with much to offer global health implementation research.

## 7. Conclusion: design matters for global health equity

This paper aimed to clarify how human-centered design may be of value to research and practice that concern global health equity. To this end we developed three contributions, by reviewing the relevant literature and reflecting on ongoing action research experiences. First, we explored the future-oriented themes of iteration and hands-on engagement that have given the design community a very different sense of disciplined practice and rigorous use of evidence than that which prevails among medical scientists. Second, with this disciplinary gulf in perspective, we surveyed the decades of work within the design community that have shaped the practice of human-centered design. Given the variety of opinion that persists among design researchers, human-centered design is best understood as an umbrella term. In particular, many associate human-centered approaches with participatory co-design, with supporting or augmenting human skills, and with attending to human values throughout the course of an iterative design and implementation process. Despite the popular excitement about human-centered design, these themes are far from universally understood among practitioners or among researchers from other disciplines. At least some degree of responsibility for this problem lies with design researchers; we have been too content to publish in disciplinary silos and to neglect the dissemination activities that would raise broader awareness of our work.

Our consideration of these themes in the design literature was not a purely academic exercise, but part of an ongoing action research effort focused on supporting a growing community designers and implementers of open source software for health. For this reason, our third contribution was to consider the value of human-centered design in terms of its relevance to global health practice. We did so by reflecting on our own experiences with over seventy digital health initiatives. This section of the paper revisited themes of iteration, participation, human skills, and human values, this time in more concrete terms that we came to understand through particular design initiatives. In light of these experiences, we emphasized that human-centered design is not restricted to building technologies or solving purely technical problems, so much as it is a way of accompanying health workers and making sense of the complex challenge of health systems strengthening in a digital age. This is not to say that the design practitioner can guarantee good outcomes; treating human-centered design as a panacea would rob it of this generative openness to complexity. Limitations notwithstanding, human-centered design has much to offer for innovation in digital health, for implementation research and for pragmatic efforts to integrate and strengthen fragmented delivery systems. Design matters for global health equity, and what is more, equity in global health matters for human-centered design.

## Notes

1 For global health and development focused resources, see: http://designforhealth.org, http://hcd4i.org, http://engagehcd.com, http://designkit.org, and http://digitalprinciples.org.

2 Google Scholar indexed just 23 publications that used the term “human-centered design” between 1978 and 1988, while 547 publications used the term between 1988 and 1998, and 3,810 the following decade.

3 See for example, Eckman, Gorski, & Mehta, 2016; Gilliam, Martins, Bartlett, Mistretta, & Holl, 2014; Gupta, Patel, Murty, Panicker, & Chen, 2015; Fotso & Fogarty, 2015; Matheson, Pacione, Shultz, & Klügl, 2015.

4 See https://www.designforhealth.org/what-is-design-for-health.

5 See https://www.nytimes.com/2003/11/30/magazine/the-guts-of-a-new-machine.html.

6 For such a definition, we would refer our readers to scholars who define design work as a situated practice and emphasize how users and stakeholders, broader social circumstances and concrete materials tend to shape design practice (Kimbell, 2011).

7 For a relevant research paper that uses the design squiggly see (Sanders & Stappers, 2008). For a longer list of appearances in academic books and the popular media, see https://thedesignsquiggle.com.

8 As is typical for open-source applications, this count is publicly viewable, see: http://github.com/medic.

9 A similar point is made by the anthropologist Lucy Suchman (1994).
